# Staged cervical osteotomy:a new strategy for correcting ankylosing spondylitis thoracolumbar kyphotic deformity with fused cervical spine

**DOI:** 10.1186/s13018-019-1146-5

**Published:** 2019-04-23

**Authors:** Tianhao Wang, Diyu Song, Guoquan Zheng, Yan Wang

**Affiliations:** 1 Southwest Hospital, Third Military Medical University, Chongqing, 400038 China; 2 Department of Orthopaedics, General Hospital of Chinese People’s Liberation Army, Fuxing Road, Beijing, 100853 China; 3 Department of Orthopaedics, The General Hospital of PLA Rocket Force, Beijing, 100088 China

**Keywords:** Ankylosing spondylitis, Deformity, Kyphosis, Thoracolumbar kyphosis, Cervical hyperlordosis, Osteotomy, Cervical osteotomy, Sagittal balance, Chin-brow vertical angle

## Abstract

**Background:**

In patients with cervical ankylosis, the chin-brow vertical angle (CBVA) should be taken into consideration. Usually, the correction of sagittal balance is sacrificed to ensure the patient has a horizontal visual field. To our knowledge, a staged osteotomy strategy for ankylosing spondylitis kyphotic deformity with an ankylosed cervical spine has not been reported before. The aim of this study was to describe a new surgical strategy with emphasis on sagittal balance and gaze angle in correction of kyphotic deformity with a rigid cervical spine in ankylosing spondylitis thoracolumbar kyphotic deformity.

**Methods:**

A 36-year-old man has severe thoracolumbar kyphosis accompanied with cervical hyperlordosis caused by ankylosing spondylitis. A two-stage surgery planning was managed. For the first stage, an interrupted two-level osteotomy was performed at the thoracolumbar area. After surgery, sagittal imbalance was corrected but the CBVA was − 21.7°. Cervical osteotomy was performed for the second stage. A flexion osteotomy was performed at C7, using anterior-posterior-anterior approaches.

**Results:**

Both sagittal imbalance and gaze angle of the patient were improved markedly. The osteotomy sites were documented fused. Complications were not observed during and after operation.

**Conclusions:**

The aim of osteotomy for ankylosing spondylitis is to reestablish sagittal balance and improve forward gaze and the visual field. A staged cervical osteotomy is an alternative to reduce cervical lordosis to obtain a normal gaze angle. An anterior-posterior-anterior approach is recommended.

## Introduction

Ankylosing spondylitis (AS) causes characteristic spinal deformity leading to impaired ability of walking, standing, and looking straight ahead in the late stages [1, 2]. Since Smith-Petersen [3] performed an opening wedge lumbar osteotomy for AS flexion deformity, more and more researches about osteotomy techniques were reported. The main purpose of corrective osteotomy is a good correction of sagittal balance, which is recommended in most of papers to be achieved by one or two-level osteotomy in the lumbar or lower thoracic spine [1–4].

Suk et al. [5] firstly noted that the chin-brow vertical angle (CBVA) was significant in the correction of AS deformity. Song et al. [6] suggested AS patients had the best satisfaction when the CBVA was between 10° and 20°. In patients with cervical ankylosis, the CBVA should be taken into consideration [7]. Usually, it is well accepted to perform a less osteotomy angle to ensure the patient’s horizontal visual field [4, 7, 8]. As a consequence, the correction of sagittal balance is sacrificed to some extent.

Although there are reports in the literature on cervical osteotomy, most of them are described to correct cervical kyphotic deformity. There were only two studies reporting cervical osteotomy for reducing lordosis. Sengupta et al. [1] reported a case of flexion osteotomy of the cervical spine for correction of iatrogenic extension deformity in ankylosis. Kose et al. [9] reported three cases of anterior closing wedge osteotomy for the treatment of cervical hyperlordosis.

To our knowledge, a staged osteotomy strategy for AS kyphotic deformity with an ankylosed cervical spine has not been reported before. This report explored a new strategy for correcting AS with thoracolumbar kyphosis and cervical ankylosis but having a normal CBVA. This deformity was corrected by two stages: an interrupted two-level osteotomy at the thoracolumbar spine for the first stage and a cervical osteotomy performed 3 months later.

## Materials and methods

### Patient information

**Fig. 1:**
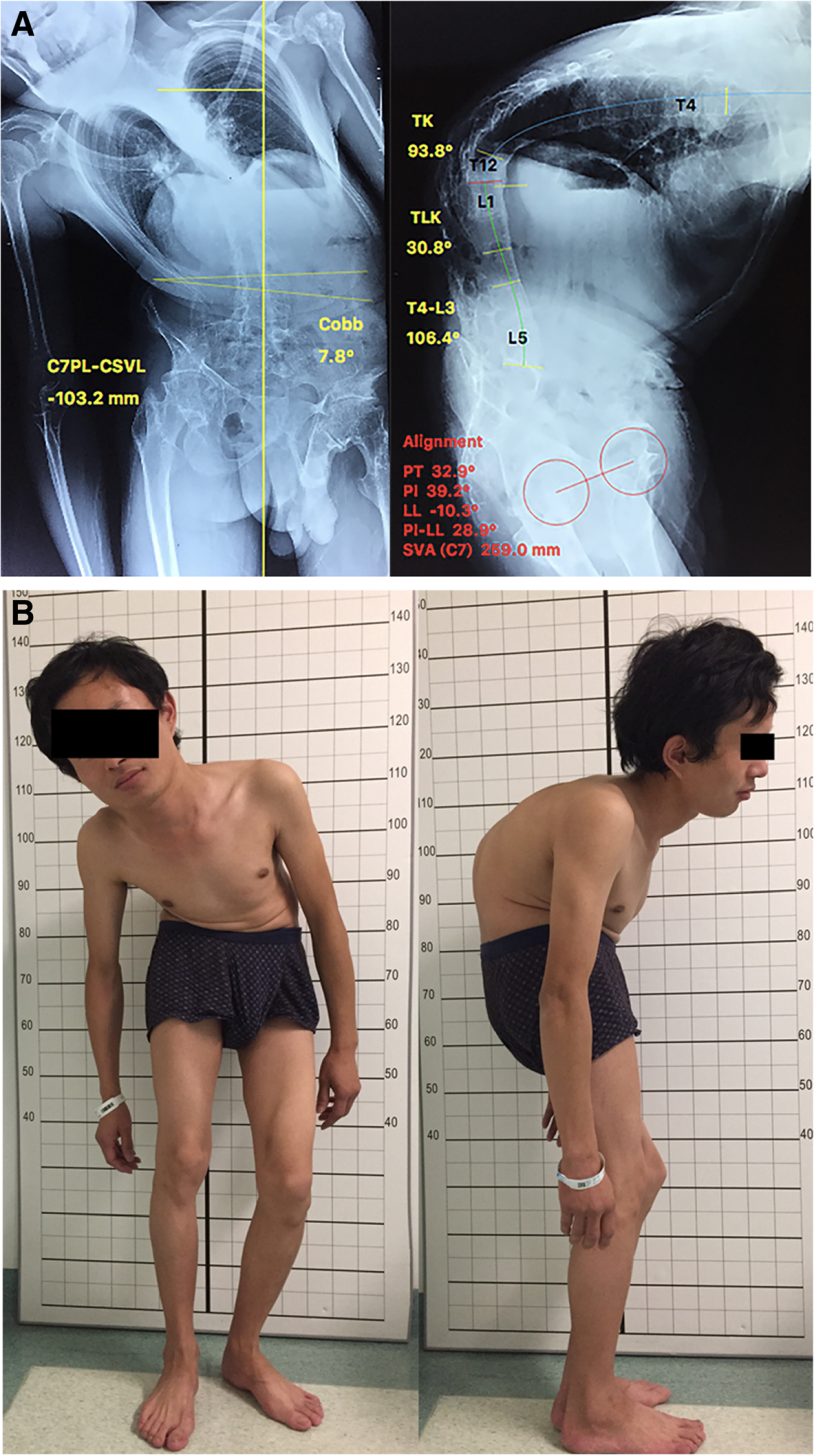
**a** Preoperative X-ray. TK, PT, PI, and SVA were 93.8°, 32.9°, 39.2°, and 259.0 mm, respectively. The total kyphosis of the spine (T4–L3) was 106.4°. **b** Preoperative clinical photographs showing the patient with thoracolumbar kyphoscoliosis. His chin-brow vertical angle was 21°

**Fig. 2:**
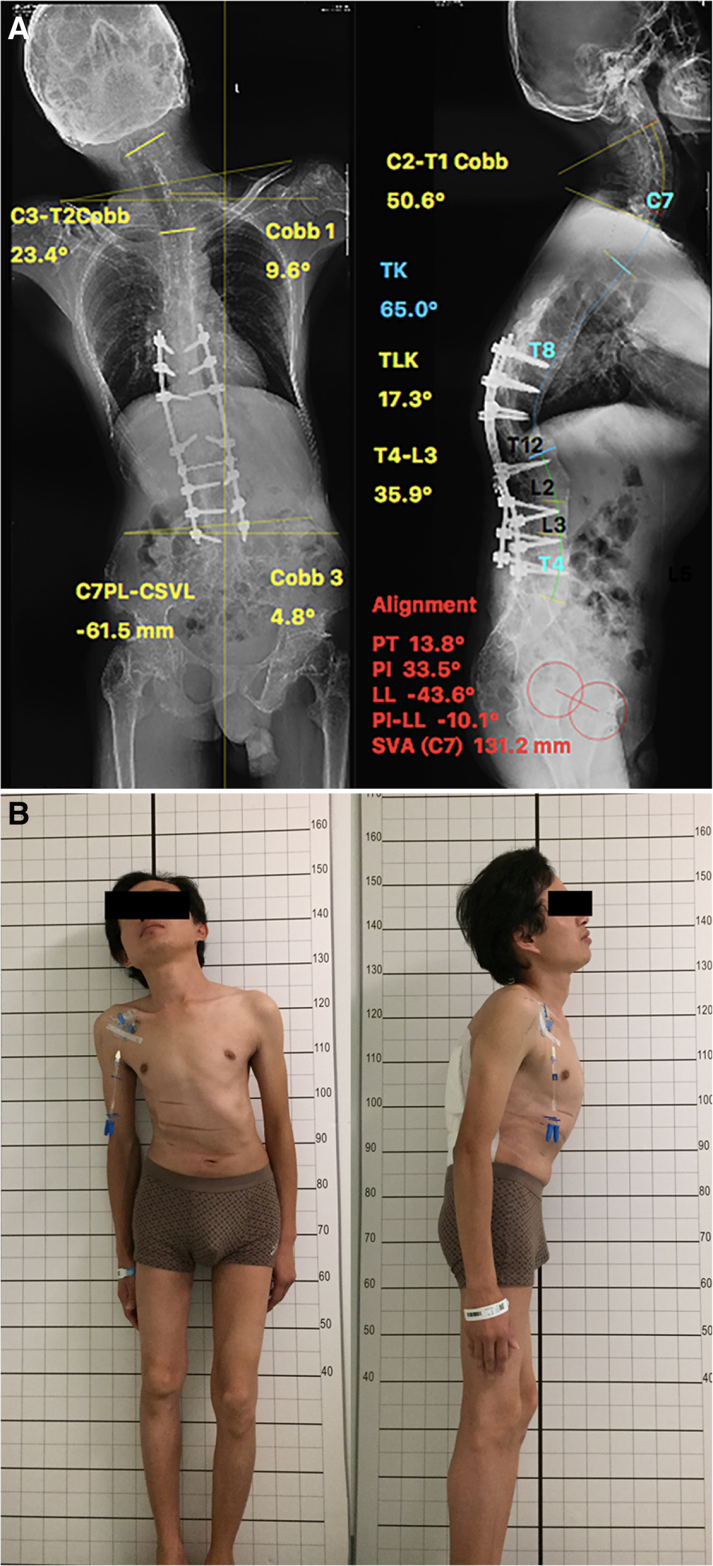
**a** Postoperative standing X-ray of the AS patient after interrupted two-level VCD at T12 and L2. The TK, PT, PI, and SVA were 65.0°, 13.8°, 33.5°, and 131.2 mm, respectively. The total kyphosis of the spine (T4–L3) was 35.9°. The Cobb angle of C2–T1 in the sagittal plane was 50.6°. The Cobb angle of C3–T2 in the coronal plane was 23.4°. **b** Postoperative photographs of the first stage showing adequate correction of kyphoscoliosis. He cannot see anything within 3 m. His CBVA was − 21.7°

A 36-year-old man with thoracolumbar kyphoscoliosis presented to our clinic. He was diagnosed with ankylosing spondylitis at the age of 16 and spine deformity which gradually progressed to a degree where it was impossible for him to stand straight. The preoperative thoracic kyphosis (TK), thoracolumbar kyphosis (TLK), lumbar lordosis (LL), and sagittal vertical axis (SVA) were 93.8°, 30.8°, − 10.3°, and 259 mm, respectively. Although having severe thoracolumbar kyphotic deformity and an unmovable neck, the patient was still able to look horizontally with a CBVA of 21°. His cervical spine was totally fused (Figs. 1 and 2).

### Surgical planning

**Fig. 3:**
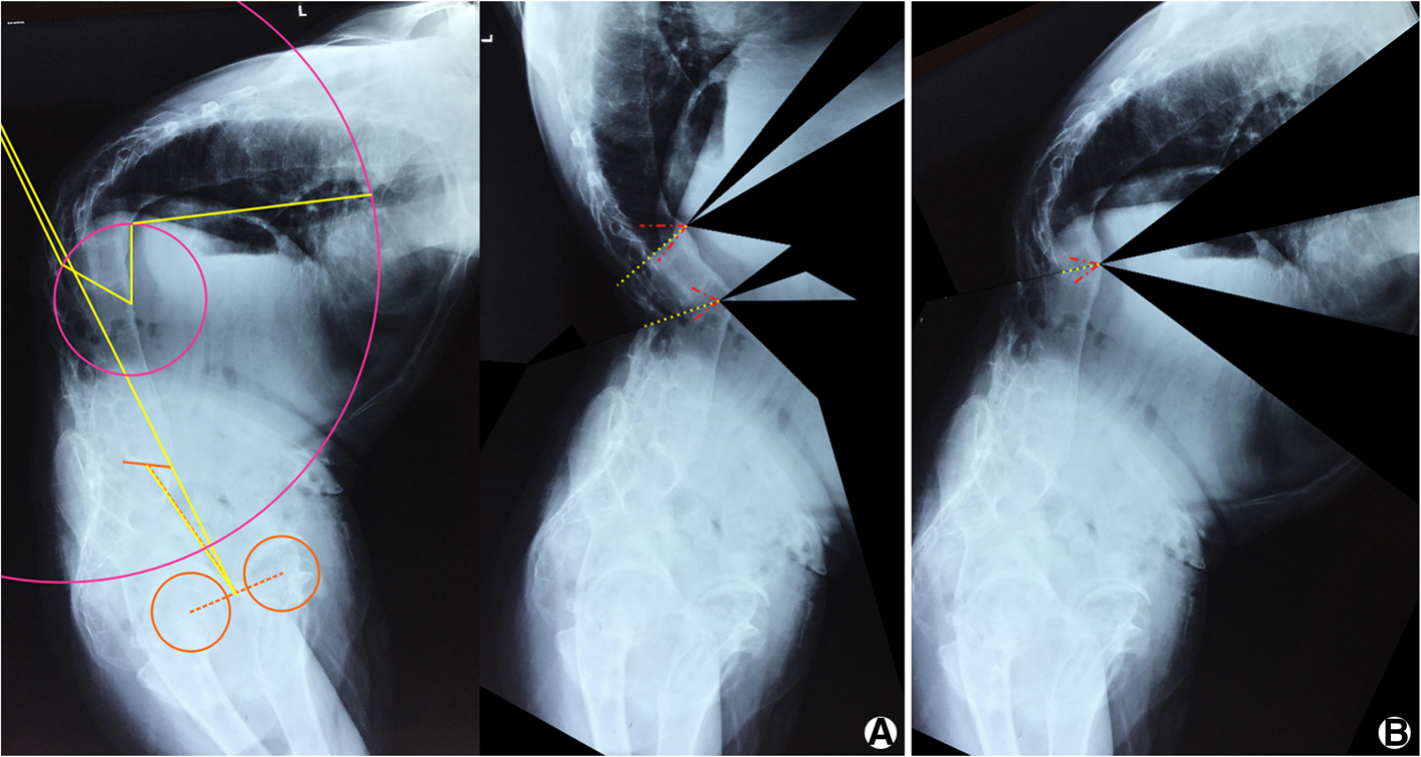
Correction angle calculated for thoracolumbar osteotomy. **a** The proper correction was 110° for this patient, but the patient will look upward after surgery. **b** If ensuring a normal CBVA, the osteotomy angle should not be larger than 49°. This degree of correction was not able to correct kyphotic deformity, and the sagittal plane was still in an imbalance condition postoperatively

A two-stage surgical plan was managed for this patient. For the first stage, the aim was to correct thoracolumbar kyphoscoliosis and restore sagittal balance without consideration of the CBVA. The required correction angle was calculated by Song’s^7^ and Zheng’s^4^ methods. The proper correction was 110° for this patient (Fig. 3). However, if we perform the osteotomy as calculated, the patient will not have a normal visual field and look upward. According to Song’s and Zheng’s research, the osteotomy angle should not be larger than CBVA + (PT − tPT). So the correction angle has to be smaller than 49°. This degree of correction was not able to correct the kyphotic deformity, and the sagittal plane was still in an imbalance condition postoperatively (Fig. 3). So we determined to make two-level osteotomies for correction of 80°. Interrupted two-level osteotomy, using the technique of vertebral column decancellation and asymmetrical osteotomy, was performed at T12 and L2 for 30° and 50°, respectively. The second-stage cervical osteotomy was planned to be performed 3 months later to make the patient have a horizontal visual field.

The main concern about the correction angle of the second cervical surgery was the CBVA. The aim of the second-stage surgery was to make the patient have a normal visual field. In this case, the CBVA before the second surgery was − 21.7°. According to Song’s research, the optimal CBVA should be 10° to 20°. Concerning the high risk of cervical osteotomy, we planned to correct the CBVA to 10° postoperatively. Therefore, a 30° osteotomy angle should be performed at the cervical spine. We measured the antero-posterior diameter of the C7 vertebra. In order to make a 30° correction angle of the wedge osteotomy, the length of anterior edge should be 1.2 cm.

### Surgical technique of cervical osteotomy

**Fig. 4:**
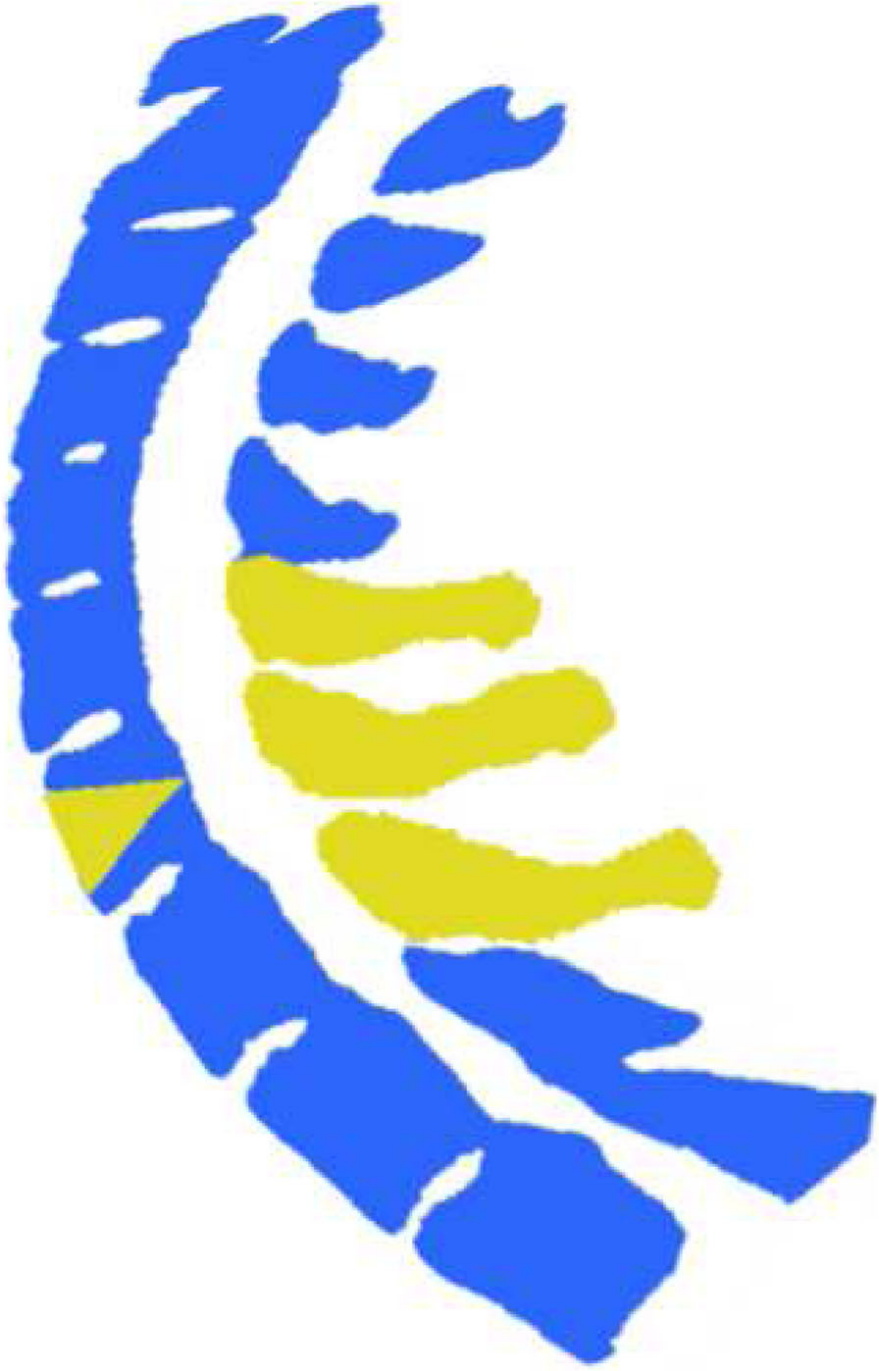
A wedge osteotomy was performed at the anterior and middle columns. Laminectomies were performed from C6 to T1. The planned osteotomy angle is 30°. The length of the anterior edge of osteotomy is 1.2 cm

Under general anesthesia and with a halo attachment stabilizing the head, the patient was placed in a supine position. A cervical anterior approach was used for the first step to perform an anterior osteotomy at C7. Considering the scoliosis on the cervicothoracic spine, a coronal angle was preserved during osteotomy. The anteriorly closing wedge osteotomy was performed using the posterior vertebral wall as a hinge (Fig. 4). Gelfoams were filled into the osteotomy space to stop the bleeding, and a gauze was put in front of the vertebrae to prevent esophageal injury.

Then the patient was placed in a prone position. Pedicle screws were placed at C4, C5, C6, T2, T3, and T4. Laminectomies were performed from C6 to T1 (Fig. 4). The pedicles of C7 were resected. Then the C7 and C8 nerve roots were explored insuring the absence of compression. A flexible iron wire was put on the left screws to avoid excessive flexion during reduction. A short titanium rod was put on the right cervical screws for temporary fixation and was held by a clamp for controlling sudden neck movement. The osteotomy was closed mainly by manipulation of the halo. When the dura tends to be extended, reduction was stopped. Posterior stabilization was achieved by a pedicle screw and titanium rod system. Then the nerve root exploration was performed again to ensure they were not compressed.

Finally, the patient was placed in a supine position again. After anterior exposure, the osteotomy site was found to be completely closed. Anterior stabilization was achieved by a cervical spine locking plate. Spinal cord monitoring was used throughout the procedure, and change of monitoring was not detected during operation.

## Results

After thoracolumbar osteotomy, TK, TLK, LL, and SVA were reduced to 65.0°, 17.3°, − 43.6°, and 131.2 mm, respectively. The C7 plumb line-center sacral vertical line (C7PL-CSVL) was reduced from 103.2 to 61.5 mm. The lordosis angle between C2 and T1 was 50.6°, and the postoperative CVBA came to be − 21.7°. Coronal Cobb’s angle between C3 to T2 was 23.4°.

**Fig. 5:**
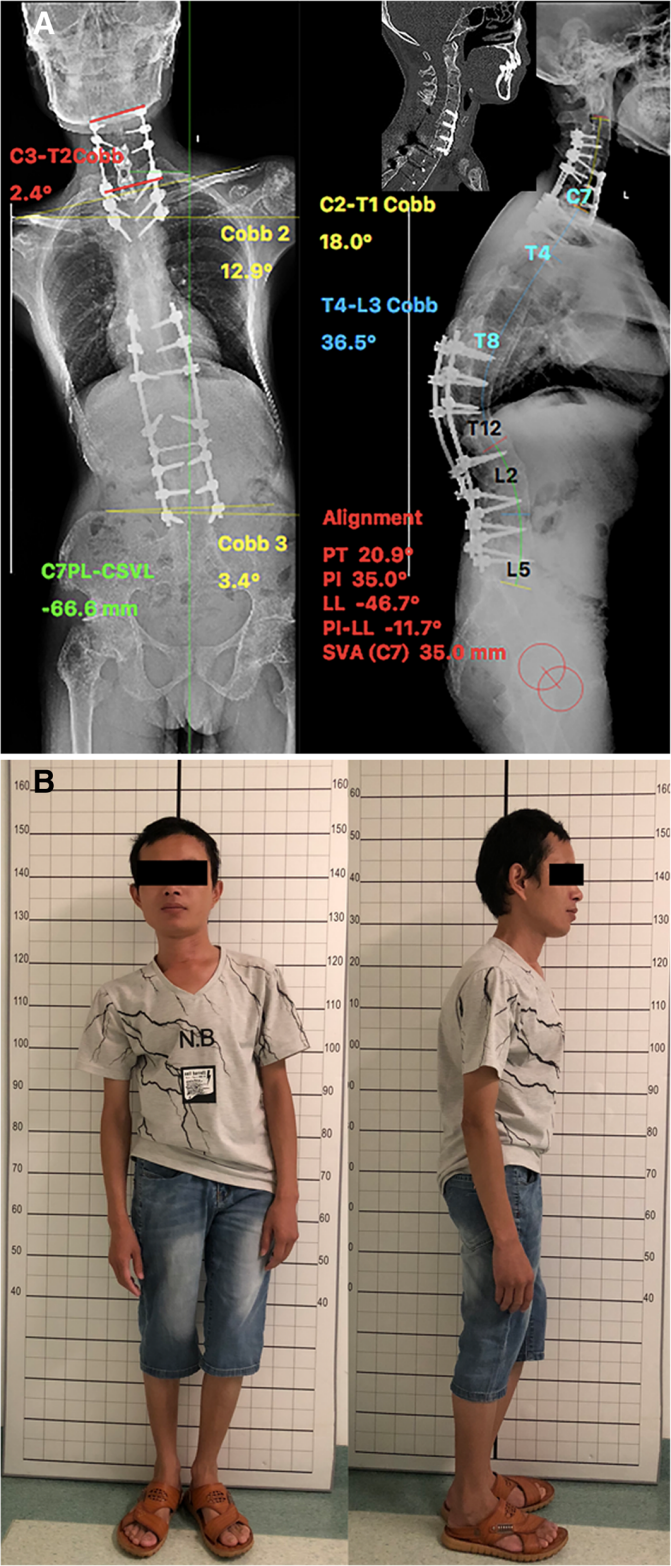
**a** Postoperative standing X-ray of the second-stage cervical osteotomy. The Cobb angle of C2–T1 in the sagittal plane was 18°. The Cobb angle of C3–T2 in the coronal plane was 2.4°. Three months after the second-stage cervical osteotomy, the CT scan of the spine showing bone healing of the osteotomy site. **b** Three months after the second-stage cervical osteotomy, the photographs show the patient with full correction of the spine. His CBVA was 3°

After the second-stage cervical osteotomy, coronal Cobb’s angle between C3 to T2 was reduced to 2.4°. The lordosis from C2 to T1 was reduced to 18°. The CBVA came to be 7.3° after the surgery. The osteotomy site was solid fused at 3 months follow-up. (Fig. 5)

Complications, such as neurological deficit and vascular injury, were not observed. The patient felt the abdominal skin and muscles tense after the first-stage surgery, but recovered 1 week later. There was no significant change in muscle strength and skin sensation after the operations.

## Discussion

There are two main goals of osteotomy to correct kyphotic deformity in AS: one is the restoration of sagittal balance and another is the restoration of forward gaze [1]. When thoracolumbar kyphosis combines with cervical ankylosis, these two main goals are not able to be achieved at the same time. Usually, a smaller angle is considered to be the ideal one for osteotomy to keep the horizontal gaze, which sacrifices the correction of sagittal imbalance to some extent [8]. The new osteotomy strategy is described in this study, and the two goals are able to be achieved together.

### Planning for restoring sagittal balance

Several osteotomy techniques have been described to restore sagittal balance for AS kyphotic deformity. To treat severe kyphotic deformity with TLK combined with loss of lumbar lordosis, a two-level osteotomy should be applied [4]. In a previous study, Song and Zheng et al. [7] chose hilus pulmonis as the center of gravity for AS TLK and put forward an accurate method for calculating the exact angle required for one-level spinal osteotomy. Zheng et al. [4] provided a further method to calculate the individualized exact angle for two-level osteotomy. In this method, theoretical individual PT is calculated by preoperative PI and is used to achieve a pelvic neutral position, and the center of gravity is designed to be shifted to a pelvic neutral positional plumb line for sagittal balance.

In the reported case, the individualized osteotomy angle was calculated. The preoperative CBVA was 21°, which was almost within the most suitable range. If an osteotomy angle of 110° was performed, the patient will not able to see anything in front of him. If considering the CBVA, the osteotomy angle should not be larger than 49° or severe kyphotic deformity and sagittal imbalance will remain consequently. Therefore, in order to correct sagittal imbalance, a compromised method was adopted. An interrupted two-level osteotomy was performed at T12 for 30° and L2 for 50° to restore sagittal balance. After surgery, SVA was reduced from 25.9 to 13.1 cm. As expected, the postoperative CBVA came to be − 21.7°. The problem of visual field was designed to be settled by the second-stage cervical osteotomy.

### Planning for cervical hyperextension osteotomy

Cervical osteotomy was a big challenge because of the difficulty and high risk of neurovacular complications [8, 10]. Performing PSO at the cervicothoracic junction is recommended to correct cervical kyphosis [10–12]. However, cases of cervical osteotomy correcting hyperlordosis were seldom reported. Sengupta et al. [1] reported a case of flexion osteotomy of the cervical spine. The author performed posterior transverse osteotomy in a right lateral decubitus position initially. And then an anteriorly based wedge was resected from C7 in the same position. An anterior cervical spine locking plate was used for achieving stabilization. Kose et al. [9] reported three cases of anterior closing wedge osteotomy for the treatment of cervical hyperlordosis. The anterior vertebral column was exposed firstly, and C7–T1 closing wedge osteotomy was performed using the disc as a hinge. Then, the posterior fixation and reduction were completed by a posterior approach. Finally, the patient was turned supine again and an anterior plate was applied.

### Approach for cervical hyperextension osteotomy

We used classical prone and supine positions and managed anterior-posterior-anterior approach osteotomy for the current case. An anterior wedge osteotomy was performed on the C7 vertebra with the hinge at the posterior vertebral wall. In the patient reported in this study, the cervical spine was fused. Therefore, although the “V”-shaped osteotomy was performed and a part of the anterior and middle columns was resected, the fused posterior column was still able to provide stability for the cervical spine. Additionally, after osteotomy, it was easy to isolate the esophagus and vertebral body, which may decrease the risk of esophageal injury during reduction. Under a cervical post approach, three steps were achieved. Firstly, implanting pedicle screws and rods provides stability after osteotomy. Secondly, decompression of the spinal cord may decrease the risk of neurological deficit. Thirdly, the spinal cord is under direct vision during reduction, which is also a benefit to avoid neurological complications. With the posterior vertebral wall closing, the spinal cord traction was limited. Also, the bone-on-bone contact provides stiffness and higher fusion rates. A stable fixation is also an important issue. We used both anterior and posterior fixation. So the third step of the anterior approach is necessary.

To achieve the goal of reducing cervical lordosis, neither the posterior-only approach nor the anterior-only approach is feasible. The posterior-only approach is not able to shorten the anterior column. It will take risks of spinal cord extension and neurological complications as well. The anterior-only approach makes it easy to perform osteotomy at the anterior and middle columns. But due to the fused posterior column, the process of reduction is hard to perform. Therefore, the anterior and posterior combined approach is required. The combined approach destroys the stability of the cervical spine for correction by osteotomy and decompression.

In most cases, making a smaller correction angle is able to ensure the patient has a horizontal visual field. However, the case we report was special. Before correcting thoracolumbar kyphosis, the patient’s CBVA was normal. Due to the fused cervical spine, once we correct the TLK, no matter how large the correction angle, the patient will look up to the sky. So there was no alternative to make a two-stage surgical planning. The current treatment strategy is suitable for severe cases like thoracolumbar kyphosis combined with cervical ankylosis. In this group of patients, correcting a smaller angle is still not able to guarantee a normal visual field, and a staged osteotomy strategy is more suitable.

## Conclusion

The two-stage surgical strategy is a successful treatment to correct AS TLKD combined with cervical ankylosis. An anterior-posterior-anterior approach closing wedge osteotomy of C7–T1 is recommended. This strategy gives consideration to both sagittal balance and visual field without sacrificing the correction of TLKD.

## Abbreviations

AS: Ankylosing spondylitis
C7PL-CSVL: C7 plumb line-center sacral vertical line
CBVA: Chin-brow vertical angle
LL: Lumbar lordosis
SVA: Sagittal vertical axis
TK: Thoracic kyphosis
TLK: Thoracolumbar kyphosis
